# Activated protein C plays no major roles in the inhibition of coagulation or increased fibrinolysis in acute coagulopathy of trauma-shock: a systematic review

**DOI:** 10.1186/s12959-018-0167-3

**Published:** 2018-06-19

**Authors:** Satoshi Gando, Toshihiko Mayumi, Tomohiko Ukai

**Affiliations:** 1 Division of Acute and Critical Medicine, Department of Anesthesiology and Critical Care Medicine, Hokkaido University Graduate School of Medicine, N15W7, Kita-ku, Sapporo, 060-8638 Japan; 2 Department of Emergency Medicine, School of Medicine, University of Occupational and Environmental Health, Kitakyushu, Japan; 3 Department of Social Medicine, Graduate School of Medicine, Osaka University, Osaka, Japan

**Keywords:** Activated protein C, Coagulation, Coagulopathy, Fibrinolysis, Plasminogen activator inhibitor-1 (PAI-1), Systematic review, Thrombin, Trauma

## Abstract

**Background:**

The pathophysiological mechanisms of acute coagulopathy of trauma-shock (ACOTS) are reported to include activated protein C-mediated suppression of thrombin generation via the proteolytic inactivation of activated Factor V (FVa) and FVIIIa; an increased fibrinolysis via neutralization of plasminogen activator inhibitor-1 (PAI-1) by activated protein C. The aims of this study are to review the evidences for the role of activated protein C in thrombin generation and fibrinolysis and to validate the diagnosis of ACOTS based on the activated protein C dynamics.

**Methods:**

We conducted systematic literature search (2007–2017) using PubMed, the Cochrane Database of Systematic Reviews (CDSR), and the Cochrane Central Register of Controlled Trials (CENTRAL). Clinical studies on trauma that measured activated protein C or the circulating levels of activated protein C-related coagulation and fibrinolysis markers were included in our study.

**Results:**

Out of 7613 studies, 17 clinical studies met the inclusion criteria. The levels of activated protein C in ACOTS were inconsistently decreased, showed no change, or were increased in comparison to the control groups. Irrespective of the activated protein C levels, thrombin generation was always preserved or highly elevated. There was no report on the activated protein C-mediated neutralization of PAI-1 with increased fibrinolysis. No included studies used unified diagnostic criteria to diagnose ACOTS and those studies also used different terms to refer to the condition known as ACOTS.

**Conclusions:**

None of the studies showed direct cause and effect relationships between activated protein C and the suppression of coagulation and increased fibrinolysis. No definitive diagnostic criteria or unified terminology have been established for ACOTS based on the activated protein C dynamics.

**Electronic supplementary material:**

The online version of this article (10.1186/s12959-018-0167-3) contains supplementary material, which is available to authorized users.

## Background

Coagulation and fibrinolysis are innate immune responses; they develop nonspecifically start following infectious and noninfectious insults, such as sepsis and trauma [1]. These responses occur at the site of the insult to limit host damage and they have roles in the compartmentalization of danger- and pathogen-associated molecular patterns, which suppress dissemination of these patterns into the circulation [1–3]. These processes are referred to as hemostasis and wound healing in trauma. During normal hemostasis, anticoagulant pathways, tissue factor pathway inhibitor (TFPI), antithrombin, protein C, and thrombomodulin restrict clot dissemination at intact sites. Protein C and thrombomodulin have important roles in controlling coagulation, fibrinolysis and inflammation via activated protein C, thrombin-activatable fibrinolysis inhibitor (TAFI) and endothelial protein C receptor (EPCR) [4, 5]. These physiological systems cannot work in severely injured trauma patients, which is associated with endothelial injury; thus, sever trauma leads to systemic thrombin generation, a condition that is called disseminated intravascular coagulation (DIC) [6].

In 2007, a condition called “acute coagulopathy of trauma-shock” (ACOTS) was newly proposed [7–9]. Another paper [10] and a review approved this hypothesis [11], and thereafter ACOTS was established as major pathophysiological concept in trauma-induced coagulopathy [12]. The pathogenesis of ACOTS is summarized as follows, based on several review articles [7, 11, 13–17]. ACOTS, in which activated protein C plays central roles, only occurs at the very early period after injury in only patients with shock and severe acidosis. Traumatic shock slows the clearance of thrombin, increasing its binding to newly expressed thrombomodulin on the normal endothelium and soluble thrombomodulin with full domains and a 100% activity in the circulation. Thrombin and thrombomodulin complexes induce the systemic production of activated protein C, which inactivates activated Factors V (FVa) and VIII (FVIIIa) and neutralizes plasminogen activator inhibitor-1 (PAI-1). This is followed by suppression of thrombin generation and an increase in the production of tissue-type plasminogen activator (t-PA). In addition, the fibrinogen levels always remain normal, indicating that less thrombin is available to cleave fibrinogen.

However, a number of researchers have expressed doubts in this theory. One group noted that the immediate massive generation of thrombin and it activation in the systemic circulation, insufficient anticoagulant pathways associated with endothelial injury and suggested that this is the main pathophysiological mechanisms of trauma-induced coagulopathy, which coincides with the definition of DIC reported by the International Society on Thrombosis and Haemostasis (ISTH) [18–23]. In addition to secondary fibrinolysis due to DIC, the accelerated release of t-PA from the injured endothelium due to traumatic shock-induced hypoperfusion, the consumption of α2-antiplasmins, and increased levels of neutrophil elastase synergistically mediate systemic fibrin (nogen) olysis. The time delay between the immediate release of t-PA from the endothelium and the expression of PAI-1 mRNA enhances fibrin (ogen) olysis immediately after injury. In addition, the massive generation of tissue factors cause fibrin (ogen) olysis. These conditions, the coexistence of DIC and pathological systemic fibrin (ogen) olysis are referred to as DIC with the fibrinolytic phenotype [24].

Another group also expressed doubts regarding the theory that ACOTS is driven by activated protein C [25–28]. They acknowledged that the currently available evidence suggests that ACOTS occurs due to the massive stimulation of thrombin generation, platelet and fibrinogen consumption, and fibrinolysis by damaged tissues, and they suggested that these data indicate a consumptive coagulopathy. Furthermore, the levels of activated protein C observed in ACOTS are far from the concentration that can cleave both platelet and plasma FVa. In plasma, PAI-1 is completely bound to vitronectin. It is therefore unlikely that activated protein C inactivates the PAI-1/vitronectin complex and leads to PAI-1 depletion. Instead, an enormous increase in the release of t-PA by endothelial cells due to shock-induced hypoperfusion is the likely cause of increased fibrinolysis. These mechanisms are very similar to DIC with the fibrinolytic phenotype; however, the lack of evidence of intravascular clot formation rules out this hypothesis according to the theory proposed by this group.

### Rationale

A recent review acknowledges that thrombin generation, hypofibrinogenemia, and endothelial dysfunction are observed in trauma-induced coagulopathies including ACOTS. This is very similar to the concept proposed by the two groups that expressed doubts in the concept of ACOTS [29]. Thus, the pathogenesis of ACOTS has been controversial for a decade since its announcement in 2007.

### Objectives

The aim of this systematic review is to address the following questions on ACOTS: 1) Does activated protein C inhibit systemic thrombin generation? 2) Does activated protein C increase fibrinolysis through the neutralization of PAI-1? and 3) Are there established diagnostic criteria for ACOTS? The participants were trauma patients (P) who had their activated protein C or activated protein C-related coagulation and fibrinolysis markers measured (E) and who had been diagnosed with ACOTS or without it (non-ACOTS) using specific definitions (C). The outcomes were evidence that activated protein C inhibits coagulation and accelerates fibrinolysis and evidence of an established diagnostic method for ACOTS (O).

## Methods

This systematic review was performed in accordance with the protocol of the Preferred Reporting Items for Systematic Review and Meta-Analysis Protocol (PRISMA-P) 2015 [30, 31]. The flow of information through the different phases of the systematic review was constructed based on the PRISMA Statement 2009 [32]. No systematic reviews have been conducted on this theme; thus, this is not an amendment of a previously completed protocol. At the point of completing the data extraction, this study did not meet the inclusion criteria of the International Prospective Register of Systematic Review (PROSPERO); thus, this systematic review was not registered.

The classifications shown in Table 1 were used in order to clarify the complicated terminology of trauma-induced coagulopathy in this systematic review [22]. Although other terms such as “acute coagulopathy of trauma” or “acute traumatic coagulopathy” etc. are now used to describe ACOTS, the original term, ACOTS, is mostly used in this systematic review. The other terms are used, as appropriate, when discussing the studies in which they are used.

**Table 1:** The classification of trauma-induced coagulopathy

1. Physiological changes
• Hemostasis and wound healing
2. Pathological changes
• Endogenously induced primary pathologies
- Disseminated Intravascular Coagulation (DIC)
• Activation of coagulation
• Insufficient anticoagulant mechanisms
• Increased fibrin (ogen) olysis (early phase)
• Suppression of fibrinolysis (late phase)
- Acute coagulopathy trauma-shock (ACOTS)
• Activated protein C-mediated suppression of coagulation
• Activated protein C-mediated increased fibrinolysis
• Exogenously induced secondary pathologies that modify DIC and ACOTS
- Anemia-induced coagulopathy
- Hypothermia-induced coagulopathy
- Acidosis-induced coagulopathy
- Dilutional coagulopathy
- Others

ACOTS is referred to by various names including (but not limited to) acute traumatic coagulopathy and acute coagulopathy of trauma, etc. Some researchers refer to ACOTS as trauma-induced coagulopathy. Adapted with permission from reference [22]

### Eligibility criteria

#### Study design

We included all studies, irrespective of whether they are prospective or retrospective in nature, with the exception of narrative reviews, current opinions, points of view, case reports, and case series.

#### Participants

We included human patients with trauma. Pediatric trauma patients were excluded.

#### Interventions

Studies that measured activated protein C or activated protein C-related coagulation and fibrinolysis markers in the blood were included. We also included studies that measured thrombin- and plasmin-related markers in the blood. Intervention studies were also included irrespective of the types of interventions. Studies that measured coagulation and fibrinolysis outside of the vessels using thromboelastgraphy or rotational thromboelastometry, which had no information on the coagulation and fibrinolysis markers in the circulation were excluded.

#### Comparators

Non-ACOTS patients and/or healthy individuals were used as controls.

#### Timing

In all of the studies that were included, the first blood sample was obtained within 12 h after injury.

Setting. The setting included emergency departments and intensive care units.

#### Language

We included articles that were written in English.

### Information sources and search strategies

We searched the following electronic bibliographic databases: PubMed, the Cochrane Database of Systematic Reviews (CSDR), and the Cochrane Central Register of Controlled Trials (CENTRAL). We also manually searched the reference lists of selected studies. The search was limited to the English literature published from January 1, 2007 to May 22, 2017. The final date of the search was May 23, 2017. The following terms were used to obtain potentially eligible articles: trauma, traumatic, injury, injuries, coagulopathy, coagulopathies, disseminated intravascular coagulation, DIC, protein C, thrombin, procoagulant, PAI-1, and fibrinolysis. The abovementioned key words were used in combination as follows: (“protein C” OR thrombin OR procoagulant OR PAI-1 OR fibrinolysis OR coagulopathy OR coagulopathies OR “disseminated intravascular coagulation” OR DIC) AND (trauma OR traumatic OR injury OR injuries).

### Data records

No specialized software programs were used for the management of the systematic review data. The extracted data were exported to Excel files (Office 2016 for Macintosh, Microsoft Japan) in the CSV format and then stored and managed. The titles and/or abstracts of studies retrieved using the search strategy and those from additional sources were screened independently by two of the review’s authors (TM and SG) to identify studies that potentially met the above-mentioned eligibility criteria. The full text of the potentially eligible studies was retrieved and independently assessed for eligibility by two of the review’s authors (TM and SG). Any disagreement between them over the eligibility of the studies was resolved through discussion with a third reviewer (TU).

### Data items

A standardized, pre-piloted form was used to extract data from the included studies in orders to assess the study quality. The information that was extracted included: the study setting; the study population and the demographics and baseline characteristics of the patients; the study methodology; the definitions of trauma-induced coagulopathy including ACOTS, and other coagulopathies or the methods by which the conditions were diagnosed; the markers of coagulation and fibrinolysis that were measured; the levels of activated protein C; evidence of the activated protein C-mediated inhibition of thrombin (or surrogate markers of thrombin generation) and PAI-1; the outcomes; and the times at which the targeted markers were measured. One of the authors (SG) extracted the data, determined the validity of the extracted data, identified discrepancies in the extracted data and identified missing data. Issues were resolved through discussion with the second (TM) and third (TU) authors.

### Outcomes and prioritization

Primary outcome. Evidence that activated protein C inhibited systemic thrombin generation by inactivating FVa and FVIIIa and evidence that activated protein C increased fibrinolysis through the neutralization of PAI-1 followed by the production of t-PA.

Secondary outcome. Evidence of established diagnostic method for ACOTS based on the activated protein C dynamics.

### Risk of bias individual studies

This systematic review did not include a meta-analysis on the outcomes and the authors did not attempt to evaluate the outcome of the included population. Thus, the assessment of the risk of bias was performed at the study level and the body of evidence was not presented.

The study quality was assessed using the Newcastle-Ottawa Scale (NOS), which scores the quality of non-randomized studies [33]. NOS has three domains based on the following: 1) the selection of the cohort, 2) the comparability of the cohorts and 3) the quality of outcome for cohort study, as well as 1) the selection of cases and controls, 2) the comparability of cases and controls and 3) the ascertainment of exposure for case-control study. NOS identifies quality with “stars”. A maximum of one star for each item within the selection and exposure/outcome categories and a maximum of two stars for comparability can be given in the NOS. The total maximum score is 9 stars. Ratings of≥ 7 were high; 4 to 6, moderate and 4 or less, low quality, were used in the present study [34].

### Data synthesis

A quantitative synthesis would not have been appropriate because of the aim of this review. A systematic narrative synthesis was provided with the information presented in the text and tables to summarize and explain the characteristics and findings of the included studies. The narrative synthesis explored the relationships and the findings both within and among the included studies.

### Meta-bias

We tried to avoid a publication biases and outcome reporting bias by submitting the results to an international journal and by clearly stating the primary and secondary outcomes.

### Confidence of cumulative estimate

We did not construct a body of evidence.

## Results

### The included studies

**Fig. 1:**
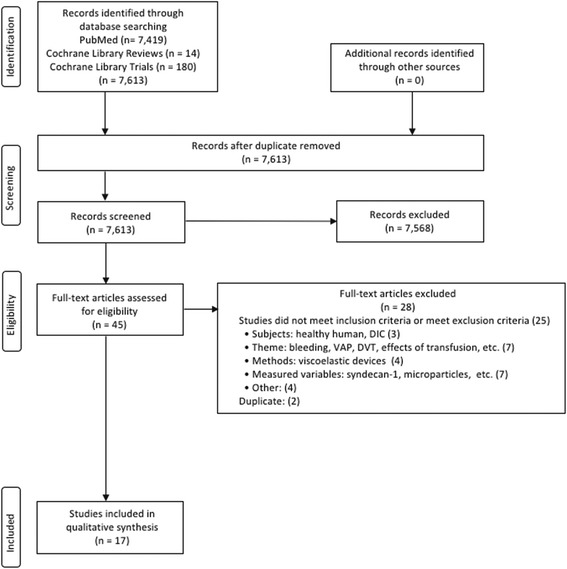
The flow of information through the different phases of the present systematic review

The flow of information through the different phases of this systematic review is presented in Fig. 1. Through these selection processes, 17 of 7613 studies met the inclusion criteria and were analyzed. Summary of the included studies are shown in Tables 2, 3, 4, 5, 6 and 7. PRISMA-P 2015 checklist is shown as Additional file 1: Table S1 [31].

**Table 2:** Studies that measure activated protein C

Reference (year)	Design	Patients (n)	Inclusion criteria	Injury Severity Score (ISS)	Sampling time (n)	Trauma-induced coagulopathy
				Mean (SD) or median (25–75% quartile)		
[35] (2011)		80	Full trauma activation	ACOTS 34(30–43) Non-ACOTS 17(10–25)	Median 68 min post injury (1)	ACOTS
[36] (2012)	Prospective cohort (s)	203	Highest-level trauma team activation	25.2 (13.8)	On arrival at ED 6,12,24 h after admission (4)	Acute traumatic coagulopathy
[37] (2012)	Prospective	132	Highest-level trauma activation system	High histone 30.5(13.0) Low histone 22.8(13.1)	Within 10 min of ED arrival 6 h after admission (2)	Traumatic injury
[38] (2013)	Prospective cohort (m)	80	Full trauma team activation	iTBI 35(32–37) sTBI + extracranial injury (sTBI/E) 25(15–26) Non-TBI 10(7–22)	Within 2 h after injury (1)	ACOTS
[39] (2013)	A subset of PROMMT	1198	Highest-level trauma activation	PTINR Coagulopathic 31.1(15.5) Non-coagulopathic 23.9(14.4) APTT Coagulopathic 35.6(16.6) Non-coagulopathic 24.6(14.0)	Not shown	Acute traumatic coagulopathy
[40] (2016)	Prospective (1)	57	Severe trauma ISS > 9, at least 1 AIS > 3	ACOTS Non-ACOTS, not shown	Within 12 h after arrival to ED (1)	ACOTS
[41] (2017)	Prospective cohort (s)	300	Full trauma activation	ATC 23(10–34) Non-ATC 9(4–22)	Within 20 min of arrival in the ED (1)	Acute traumatic coagulopathy
[42] (2013)	Prospective (s)	57	Severe trauma ISS > 9, at least 1 AIS > 3	ACOTS Non-ACOTS, not shown	Within 12 h after arrival to ED (1)	ACOTS

**Table 3:** Studies that measure activated protein C

Reference (year)	Diagnosis	Control	Normal control	Activated protein C Methods	Thrombin Surrogate markers	PAI-1
[35] (2011)	APTT and/or PTINR above normal reference	Non-ACOTS	No	Yes ELISA	Yes PF1 + 2, TAT	Yes
[36] (2012)	PTINR> 1.3	No	No	Yes Enzyme capture assay (banzamidine)	No	No
[37] (2012)	No	Low histone	No	Yes Enzyme capture assay (banzamidine)	No	No
[38] (2013)	APTT and/or PTINR above normal reference	NA	No	Yes ELISA	Yes PF1 + 2, TAT	Yes
[39] (2013)	PT INR 1.3, APTT> 35 s.	Non-coagulopathic	No	Yes Not shown	No	No
[40] (2016)	PT ratio > 1.2	Normal control Non-ACOTS	Healthy volunteer	Yes ELISA	No	No
[41] (2017)	Amplitude of clot at 5 min by ROTEM	Non-ATC	No	Yes Enzyme capture assay (benzamidine)	Yes PF1 + 2	Yes
[42] (2013)	PT ratio > 1.2	Normal control Non-ACOTS	Healthy volunteer	Yes Prothrombinase activity	Yes Soluble fibrin	No

**Table 4:** Studies that measure activated protein C

Reference (year)	Main results
[35] (2011)	ACOTS showed lower levels of protein C, and higher levels of sTM and D-dimer however, there were no differences in the levels of activated protein C, PF1 + 2, TAT, t-PA, or PAI-1 between ACOTS and non-ACOTS. In ACOTS, activated protein C showed no correlation with PF1 + 2. ACOTS showed consumption coagulopathy.
[36] (2012)	Patients with ISS > 15 and BD > − 6 showed high activated protein C and lower protein C levels. Activated protein C > 6 ng/mL was associated with prolonged PT, APTT, low FV and FVIII activities, and high levels of t-PA and D-dimer. Markers of thrombin generation, sTM and PAI-1 were not measured. Neither activated protein C nor protein C levels were shown in patients with acute traumatic coagulopathy (defined as PTINR> 1.3).
[37] (2012)	The high histone group showed higher ISS, and activated protein C, t-PA, and D-dimer levels. The markers of thrombin generation, sTM and PAI-1 were not measured. There were no differences between high and low histone groups with regard to the levels of protein C, AT, or BD, or the FVII, FV and FVIII activities.
[38] (2013)	The sTBI/E group showed high ISS and a high incidence of ACOTS. The sTBI/E groups showed decreased protein C and AT levels, and increased D-dimer and sTM levels. There were no differences in the levels of BD, activated protein C, PF1 + 2, TAT, t-PA, or PAI-1 among the three groups.
[39] (2013)	PTINR-based coagulopathy: The coagulopathy group showed higher activated protein C levels and lower protein C levels but there were no differences in the D-dimer levels. Both protein C and activated protein C were independent predictors of coagulopathy. APTT-based coagulopathy: Coagulopathy was associated with increased activated protein C and D-dimer levels and decreased protein C levels. There were inconsistencies between PTINR- and APTT-based coagulopathy. Factors V, VII, and VIII were decreased in both types of coagulopathy.
[40] (2016)	Activated protein C levels were more decreased in ACOTS than controls and non-ACOTS. ACOTS showed the same results as DIC.
[41] (2017)	ATC showed higher activated protein C, PF1 + 2, PAP and D-dimer levels and lower protein C, AT and fibrinogen levels in comparison to non-ATC. Activated protein C reduced the Factors V and VIII activities and the fibrinogen levels in a dose-dependent manner, while the thrombin generation capacity was preserved. The PAI-1 levels did not differ between patients with low and high activated protein C levels.
[42] (2013)	ACOTS showed normal prothrombinase activity, increased soluble fibrin and sTM, and decreased AT in comparison to normal controls and non-ACOTS. The results related to ACOTS coincided with those of DIC.

*ACOTS* acute coagulopathy trauma shock, *AIS* abbreviated injury scale, *APTT* activated partial thromboplastin time, *AT* antithrombin, *ATC* acute traumatic coagulopathy, *BD* base deficit, *E* extracranial injury, *ED* emergency department, *DIC* disseminated intravascular coagulation, *ISS* injury severity score, *iTBI* isolated traumatic brain injury, *m* multicenter, *PAI-1* plasminogen activator inhibitor-1, *PAP* plasmin α2-antiplasmin complex, *PF1 + 2* prothrombin fragment 1 + 2, *PT* prothrombin time, *PTINR* prothrombin time international normalized ratio, *ROTEM* rotational thromboelastometry, *s* single center, *SD* standard deviation, *sTBI* severe traumatic brain injury, *sTM* soluble thrombomodulin, *TAT* thrombin antithrombin complex, *t-PA* tissue-type plasminogen activator

**Table 5:** Studies that measure activated protein C-related coagulation and fibrinolysis markers

Reference (year)	Design	Patients (n)	Inclusion criteria	Injury Severity Score (ISS)	Sampling time (n)	Trauma-induced coagulopathy
				Median (25–75% quartile)		
[8] (2007)	Prospective cohort (s)	208	Full trauma team activation	17(9–26)	Arrival in the trauma room (1)	Acute traumatic coagulopathy
[9] (2007)	Prospective cohort (s)	39	Traumatic brain injury	24(14–30)	Arrival in the trauma room (1)	Coagulopathy
[10] (2008)	Prospective cohort (s)	208	Major trauma	17(9–26)	Immediately on admission to ED (1)	Acute coagulopathy of trauma
[43] (2009)	Prospective (s)	42	Initial blood sample collected within 1 h	ACT Non-ACT, not shown	Within 1 h of hospital presentation (1)	Acute coagulopathy of trauma
[44] (2010)	Prospective (s)	58	Not shown	With coagulopathy Without coagulopathy, not shown	Within 1 h of arrival at hospital	Coagulopathy
[45] (2011)	Prospective cohort (s)	334	Severe polytrauma ISS > 15	ISS 15–20, 30–50, > 50	Immediately after ED admission (1)	Trauma-induced coagulopathy
[46] (2013)	Prospective cohort (s)	303	Trauma team activation	Fibrinolytic activity by PAP and ML Normal 6(1–10) Moderate 17(9–28) Severe 25(17–38)	Within 20 min of arrival in the ED (1)	None
[47] (2014)	Prospective cohort (s)	163	Highest-level trauma team activation	Fibrinolytic activity by PAP Normal 9(2–16) Moderate 21(13–25) Severe 28(17–35)	On admission and prior to administration blood product (1)	None
[48] (2016)	Prospective cohort (s)	72	Highest-level trauma team activation	Hyperfibrinolytic by rapid TEG 33(22–41)	The earliest possible time point after injury (1)	Trauma-induced coagulopathy

**Table 6:** Studies that measure activated protein C-related coagulation and fibrinolysis markers

Reference (year)	Diagnosis	Control	Normal control	Thrombin (surrogate)	PAI-1
[8] (2007)	No	No	No	Yes	Yes
[9] (2007)	No	No	No	Yes	Yes
[10] (2008)	No	No	No	Yes	Yes
[43] (2009)	PT > 18 s (PTINR> 1.5)	Non-ACT	Healthy volunteer	Yes	No
[44] (2010)	ISTH DIC	Without coagulopathy	Healthy volunteer	Yes	No
[45] (2011)	NA	ISS 15–29	No	Yes	No
[46] (2013)	NA	Normal fibrinolytic activity	No	Yes	Yes
[47] (2014)	NA	Normal fibrinolysis	No	No	Yes
[48] (2016)	No	Healthy volunteer	Healthy volunteer	No	Yes

**Table 7:** Studies that measure activated protein C-related coagulation and fibrinolysis markers

Reference (year)	Main results
[8] (2007)	PF1 + 2 increased as the ISS increased. In the presence of increased BD, protein C fell with increasing levels of PF1 + 2 and sTM. Low protein C was associated with low PAI-1 and increased t-PA and D-dimer levels. These changes were associated with prolonged PT and APTT.
[9] (2007)	Increasing ISS and BD were associated with high PF1 + 2, sTM levels and low levels of protein C levels. Brain injury and increased BD resulted in high t-PA and D-dimer levels. None of the results included the PAI-1 levels. These changes were associated with prolonged PT and APTT.
[10] (2008)	PF1 + 2 increased with increased ISS. Protein C fell with increasing levels of sTM. t-PA was increased in patients with BD > − 7.7; this was unrelated to PF1 + 2. The t-PA and D-dimer levels decreased in parallel with increases in PAI-1.
[43] (2009)	ACT patients had lag times that was 68% shorter and peak thrombin generation was three-fold higher in comparison to normal patients, indicating the presence of circulating procoagulants that were capable of initiating systemic coagulation. Increased systemic thrombin generation was associated with slower inhibition of thrombin generation and decreased antithrombin levels.
[44] (2010)	Patients with coagulopathy had higher procoagulant activity, tissue factor-like activity and D-dimer in comparison to normal controls and patients without coagulopathy. In patients with coagulopathy, 79% of the procoagulant activity was due to tissue factor-like activity, which was higher than that in patients without coagulopathy.
[45] (2011)	Patients with higher ISS (30–50 and > 50) showed extremely elevated PF1 + 2, TAT, and lower AT levels, which were associated with increased BD. Patients with higher ISS also showed lower platelet counts and fibrinogen levels, and prolonged PT and APTT.
[46] (2013)	In patients with severe fibrinolytic activity, the PAP, t-PA and D-dimer levels were higher than in patients with normal and moderate fibrinolysis. However, the PAI-1 levels did not change in association with changes in t-PA, D-dimer, and PAP levels. Patients with severe fibrinolytic activity also showed high PF1 + 2 and low AT levels. The PAP and D-dimer levels were increased in line with increases in the BD.
[47] (2014)	Severe fibrinolysis was associated with high active and total t-PA and a reduction of active and total PAI-1. Increased active t-PA and reduced active PAI-1 were both associated with fibrinolysis, as measured by PAP.
[48] (2016)	The total PAI-1 levels in hyperfibrinolytic trauma and healthy controls were equal; however, the levels of active PAI-1 were higher than in controls. The ratio of active to complexed PAI-1/t-PA was lower in hyperfibrinolytic trauma than in controls. Conversely, both total t-PA and active t-PA were higher than in healthy controls. Massive t-PA release overwhelms free PAI-1. There is no PAI-1 degradation.

*ACT* acute coagulopathy of trauma, *APTT* activated partial thromboplastin time, *AT* antithrombin, *BD* base deficit, *DIC* disseminated intravascular coagulation, *ED* emergency department, *ISTH* International Society on Thrombosis and Haemostasis, *m* multicenter, *ML* maximum clot lysis, *PAI-1* plasminogen activator inhibitor-1, *PAP* plasmin α2-antiplasmin complex, *PF1 + 2* prothrombin fragment 1 + 2, *PT* prothrombin time, *PTINR* prothrombin time international normalized ratio, *s* single center, *sTM* soluble thrombomodulin, *TAT* thrombin antithrombin complex, *TEG* thromboelastography, *t-PA* tissue-type plasminogen activator

### Study quality

All of the studies included were prospectively performed, but there were no randomized controlled studies. A clear control group or non-exposed cohort (non-ACOTS) had been established in seven of the studies, and data from normal healthy controls were presented in five studies. The overall quality of studies was low, with a mean score of 3.5 and a range of 1 to 7. In particular, the quality of the cohort studies was very low, with mean NOS score of 2.2. The assessment of the study quality using the NOS is shown in Additional file 2: Table S2.

### Activated protein C (Tables 2, 3 and 4)

Only 7 studies directly measured the activated protein C level [35–41] and 1 study measured the prothrombinase activity as surrogate marker of function of activated protein C [42]. Thrombin generation was assessed in 4 studies and PAI-1 was measured in 3 studies. Three studies simultaneously measured activated protein C, thrombin generation, and PAI-1 [35, 38, 41].

The activated protein C levels of the ACOT and non-ACOT subjects did not differ to a statistically significant extent [35] or the activated protein C levels in the ACOTS were significantly decreased in comparison to the non-ACOTS [40]. The former study showed no differences in the levels of prothrombin fragment 1 + 2 (PF1 + 2), thrombin and antithrombin complex (TAT) or PAI-1 between ACOTS and non-ACOTS. Significant differences in the Injury Severity Score (ISS) and the diagnosis of ACOTS did not affect the levels of activated protein C, PF1 + 2, TAT, or PAI-1 [38]. Normal prothrombinase activity in ACOTS was associated with increased levels of soluble fibrin, a direct marker of thrombin generation and its activation [42].

Three studies confirmed elevated activated protein C levels in patients with ISS of > 15 and a base deficit of < 6, in patients with histone levels of > 50 AU, or in patients with prothrombin time international normalized ratio (PTINR)-based and activated partial thromboplastin time (APTT)-based coagulopathy. However, no results on the relationships between activated protein C and thrombin generation or activated protein C and PAI-1 were presented [36, 37, 39]. The higher levels of activated protein C in acute traumatic coagulopathy, in comparison to non-acute traumatic coagulopathy, were associated with the significant elevation of PF1 + 2 [41]. This study also confirmed that activated protein C levels did not affect the levels of PAI-1.

Finally, activated protein C is well known to be immediately inactivated by protein C inhibitor, α1-antitrypsin, α2-antiplasmin, and α2-macroglobulin in the circulation. Therefore, the methods of measurement are very important to consider when discussing the level and function of activated protein C in the circulation. As shown in Tables 2, 3 and 4, different methods of measuring activated protein C were used in eight studies, which may have affected the results of these studies. The validation of measurement methods used in each study is mandatory.

### Thrombin generation and PAI-1 (Tables 5, 6 and 7)

Although they did not measure the levels of activated protein C, 9 studies investigated activated protein C-related markers, thrombin generation and PAI-1 [8–10, 43–48]. Three studies hypothesized, based on low protein C levels, that activated protein C contributes to the suppression of thrombin generation and hyperfibrinolysis via the inactivation of FVa and FVIIIa and the consumption of PAI-1, respectively [8–10]. However, none of the three studies showed direct evidences to support these hypotheses.

In contrast, systemic thrombin generation or tissue factor activity in the circulation were confirmed in acute coagulopathy of trauma [43], traumatic coagulopathy [44], and severely injured trauma patients [45, 46]. In patients with severe fibrinolysis, an increased t-PA level was not associated with any decreases in the PAI-1 levels. In addition, the PAI-1 levels were similar among the patients with normal, moderate, and severe fibrinolysis [46]. With regards to the mechanism underlying the increase in active t-PA without concomitant increase in active PAI-1 [47], one study suggested that a massive t-PA release overwhelms the free PAI-1 and that the degradation of PAI-1 by activated protein C is not responsible for increased fibrinolysis [48].

### The definition and diagnostic criteria of ACOTS

As Tables 2, 3, 4, 5, 6 and 7 show, no unified terminology, clear definition, or internationally agreed upon diagnostic criteria for ACOTS were used in the selected studies, which were published after ACOTS became an established pathophysiological condition of trauma-induced coagulopathy [11, 12].

## Discussion

Activated protein C has both anticoagulant and profibrinolytic effects through the proteolytic inactivation of FVa and FVIIIa and through the activation of TAFI and the neutralization of PAI-1 [4, 5, 49]. Although these activities are important in normal physiological hemostasis at the injured site, the suggestion that the same properties are systemically active in pathological conditions such ACOTS is in doubt. Campbell et al. [50] suggested that the levels of activated protein C in ACOTS were insufficient to inactivate platelet and plasma FVa. Another in vitro study confirmed that 300 to 2000 ng/mL of activated protein C was needed to suppress activities of FV and FVIII, and to prolong PT and APTT [51]. These levels were extremely high in comparison to those observed in clinical studies, which indirectly shows that pathomechanisms of ACOTS are not robust [36, 37, 39].

Only 2 studies simultaneously compared the activated protein C and thrombin generation levels between ACOTS (acute traumatic coagulopathy) and non-ACOTS (non-acute traumatic coagulopathy) [35, 41]. Thrombin generation (PF1 + 2 or TAT) was preserved without changes in the activated protein C levels [35] or was significantly increased with high activated protein C levels being observed [41]. One study confirmed normal prothrombinase activity with markedly elevated thrombin generation [42]. Importantly, in studies that measured activated protein C levels, the levels were reported to be inconsistently normal, decreased, or elevated [35–42]. Although the activated protein C levels were not measured, the clinical studies consistently showed significant systemic thrombin generation in ACOTS and severely injured trauma patients [43–46]. These results suggest that - irrespective of activated protein C levels - systemic thrombin generation is increased immediately after trauma, especially in ACOTS and in patients with severe trauma.

Two studies simultaneously compared the activated protein C and PAI-1 levels between ACOTS (acute traumatic coagulopathy) and non-ACOTS (non-acute traumatic coagulopathy) [35, 41]. There were no differences in the levels of PAI-1 between patients with and without ACOTS or acute traumatic coagulopathy, irrespective of the activated protein C levels. Another study confirmed the same results in ACOTS with severe head and neck, and extracranial injuries [38]. Although the activated protein C levels was not measured, severe fibrinolysis, evidenced by increased levels of plasmin and α2-antiplasmin complex and t-PA, was not associated with decreased PAI-1 levels [46]. One study clearly demonstrated that the decrease in the PAI-1 levels in patient with trauma-induced coagulopathy was driven by an increase in t-PA, rather than by the degradation of PAI-1 by activated protein C [48]. These results suggest that activated protein C is highly unlikely to neutralize PAI-1.

A PT ratio of > 1.2 was announced as a clinically relevant definition of acute traumatic coagulopathy [52]. However, only two studies used this as diagnostic criteria for ACOTS [40, 42]. As shown in Tables 2, 3, 4, 5, 6 and 7, various criteria were used to diagnose coagulopathy in the studies that were selected for this systematic review; furthermore, various names were used to refer to the conditions. This makes the collection of uniform patients group very difficult. As a consequence, the evidence to support a uniform concept of ACOTS is very fragile.

The main pathological definition of ACOTS is the activated protein C-mediated inactivation of FVa and FVIIIa, which stops the generation of thrombin. Thus, the APTT that represents coagulation pathway including FV and FVIII, but not PT, is considered to be more suitable for the diagnosis of ACOTS [20]. The lack of a clear pathological definition and diagnostic criteria based on activated protein C dynamics leads to these authors to be skeptical of the existence of the condition referred to as ACOTS.

This systematic review failed to establish hypotheses or complete the aims stated in the Objective section.

## Conclusions

This systematic review demonstrated that there have been no studies that showed the direct cause and effect relationships between activated protein C and the suppression of systemic thrombin generation or between activated protein C and increased fibrinolysis via the neutralization of PAI-1. Systemic thrombin generation was always increased and increased fibrinolysis was observed independently from the PAI-1 levels in patients with ACOTS. The lack of a clear definition and diagnostic criteria, and various terminologies that are used to refer to ACOTS may be one of the reasons for these results. This systematic review denies pathophysiological definition of ACOTS that was proposed in 2007 and concludes that the condition that is referred to as “ACOTS” based on activated C dynamics unlikely to exist.

## Key messages

- None of the studies searched in our systematic review showed direct cause and effect relationships between activated protein C and the suppression of coagulation and increased fibrinolysis in ACOTS.
- The lack of a clear definition and diagnostic criteria, and various terminologies that are used to refer to ACOTS may be one of the reasons for these results.

## Additional files

Additional file 1: **Table S1.** PRISMA-P 2015 checklist: recommended items to address in a systematic review protocol. (PDF 189 kb)

Additional file 2: **Table S2.** Assessment of study quality using the NOS system. (PDF 102 kb)

## Abbreviations

ACOTS: Acute coagulopathy of trauma-shock (ACOTS)
ACT: Acute coagulopathy of trauma
AIS: Abbreviated injury scale
APTT: Activated partial thromboplastin time
AT: Antithrombin
ATC: Acute traumatic coagulopathy
BD: Base deficit
DIC: Disseminated intravascular coagulation
ED: Emergency department
EPCR: Endothelial protein C receptor
FDP: Fibrin/Fibrinogen degradation products
FgDP: Fibrinogen degradation products
FVa: Activated Factor V
FVIIIa: Activated Factor VIII
ISS: Injury Severity Score
ISTH: International Society on Thrombosis and Haemostasis
iTBI: Isolated traumatic brain injury
MAP: Mean arterial pressure
NOS: Newcastle-Ottawa Scale
PAI-1: Plasminogen activator inhibitor-1
PAP: Plasmin α2-antiplasmin complex
PF1+2: Prothrombin fragment 1+2
PRISMA-P: Preferred Reporting Items for Systematic Review and Meta-Analysis Protocol
PROSPERO: International Prospective Register of Systematic Reviews
PT: Prothrombin time
PTINR: Prothrombin time international normalized ratio
ROTEM: Rotational thromboelastometry
SD: Standard deviation
sTBI: Severe traumatic brain injury
sTM: Soluble thrombomodulin
TAFI: Thrombin-activatable fibrinolysis inhibitor
TAT: Thrombin and antithrombin complex
TEG: Thromboelastography
TFPI: Tissue factor pathway inhibitor
t-PA: Tissue-type plasminogen activator
