# Supplementary material for: Activated protein C plays no major roles in the inhibition of coagulation or increased fibrinolysis in acute coagulopathy of trauma-shock: a systematic review
Source: Thromb J. 2018 Jun 19;16:13. doi: 10.1186/s12959-018-0167-3 (PMC6006835; doi:10.1186/s12959-018-0167-3)
Supplement: Supplementary file 2 — Table S2. Assessment of study quality using the NOS system. (PDF 102 kb) [file 12959_2018_167_MOESM2_ESM.pdf]

Supplement Table 2 Assessment of study quality using the Newcastle-Ottawa quality assessment scale

| | Selection | | | | Comparability | Outcome | | | Total score |
|---|---|---|---|---|---|---|---|---|---|
| Cohort study | Cohort is repersentative of a general adult trauma population | ACOTS and non-ACOTS were selected from the same population | Acertainment of exposure (data were obtained from secure record) | Demonstration that outcome of interst was not present at start of study | Comparability of cohorts on the basis of the design or analysis | Assessment of outcomes | Follow up long enough for outcomes to occur | Adequacy of follow up of cohorts | |
| 8 (2007) | * | | * | | | | | | 2 |
| 9 (2007) | | | * | | | | | | 1 |
| 10 (2008) | * | | * | | | | | | 2 |
| 36 (2012) | * | | * | | | | | | 2 |
| 38 (2013) | | | * | | | | | | 1 |
| 39 (2013) | * | * | * | | * | | | | 4 |
| 41 (2017) | * | * | * | | * | | | | 4 |
| 45 (2011) | * | | * | | | | | | 2 |
| 46 (2013) | * | | * | | | | | | 2 |
| 45 (2014) | * | | * | | | | | | 2 |
| 48 (2016) | * | | * | | | | | | 2 |
| | Selection | | | | Comparability | Exposure | | | Total score |
| Case-control styudy | Adequate ACOTS definition | Representativeness of the ACOTS | Selection of non-ACOTS | Definition of ACOTS | Comparability of cohorts on the basis of the design or analysis | Acertainment of exposure (data were obtained from secure record) | Same method of ascertainment for ACOTS and non-ACOTS | Non-response rate | |
| 35 (2011) | * | * | * | * | * | * | * | | 7 |
| 37 (2012) | | | | | | * | | | 1 |
| 40 (2016) | * | | * | * | * | * | * | | 7 |
| 42 (2013) | * | * | * | * | * | * | * | | 7 |
| 43 (2009) | * | * | * | * | * | * | * | | 7 |
| 44 (2010) | * | * | * | * | * | * | * | | 7 |

A maximum2 stars can be allotted in Comparablility category. ACOTS is used as a representative terminology of other terminologies such as acute traumatic coaguloapthy and acute coagulopathy of trauma defined in idividual studies.
